# Supplementary material for: Structure of the activated Edc1-Dcp1-Dcp2-Edc3 mRNA decapping complex with substrate analog poised for catalysis
Source: Nat Commun. 2018 Mar 20;9:1152. doi: 10.1038/s41467-018-03536-x (PMC5861098; doi:10.1038/s41467-018-03536-x)
Supplement: Supplementary file 1 — Supplementary Information(PDF 2281 kb) [file 41467_2018_3536_MOESM1_ESM.pdf]

SUPPLEMENTARY INFORMATION

Structure of the activated Edc1-Dcp1-Dcp2-Edc3 mRNA decapping complex with substrate analog poised for catalysis

Mugridge *et al*.

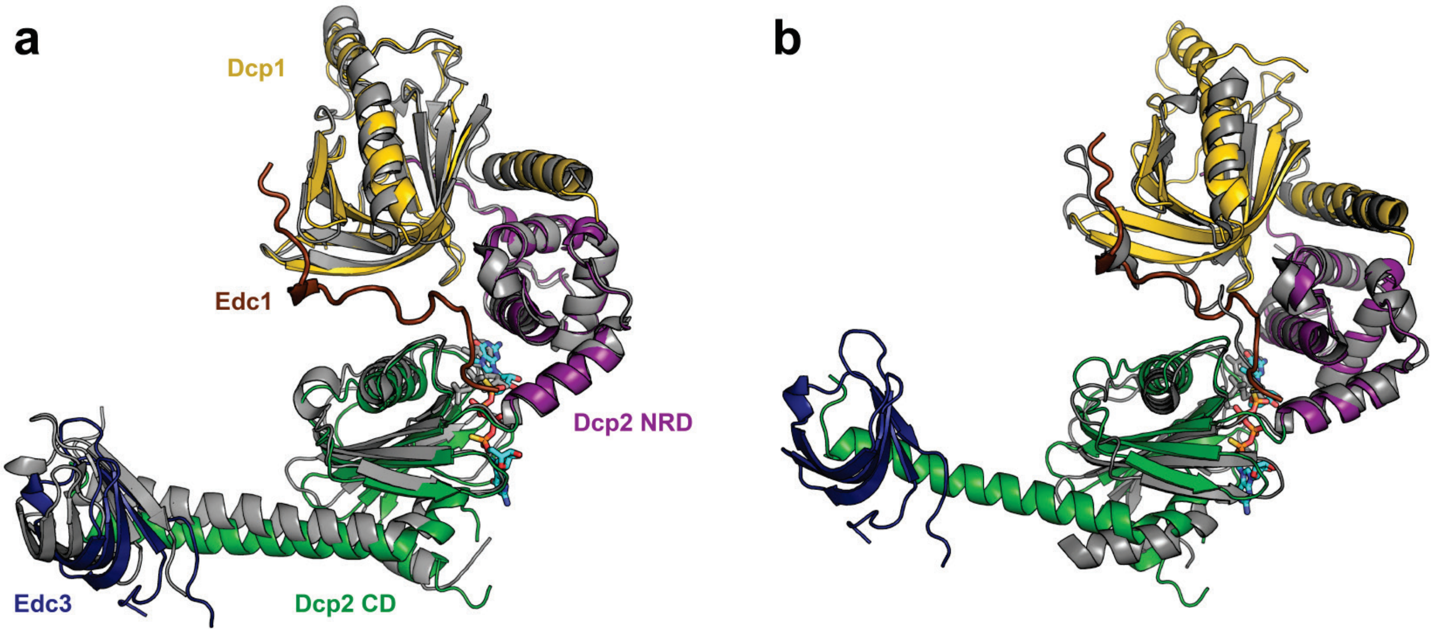

Supplementary Figure 1. Alignment of recent structures of Dcp1-Dcp2 in the active conformation.

(a) Alignment of *Kl* Dcp1-Dcp2-Edc3-m^{7}GDP structure (PDB 5LOP, gray) with *Kl* Edc1-Dcp1-Dcp2-Edc3-substrate analog structure from this paper (PDB 6AM0, colored). Dcp2 NRD (residues 8-97) was used to align the two structures shown above (backbone RMSD is 0.78 Å for NRD alignment over 360 atoms). Backbone RMSD for alignment of the whole Dcp1-Dcp2-Edc3 complex is 1.8 Å over 1695 atoms. **(b)** Alignment of *Sp* Edc1-Dcp1-Dcp2-m^{7}GDP (PDB 5N2V, gray) with *Kl* Edc1-Dcp1-Dcp2-Edc3-substrate analog structure from this paper (PDB 6AM0, colored). Dcp2 NRD (*Kl* residues 8-97, *Sp* residues 7-94) was used to align the two structures shown above (backbone RMSD is 1.65 Å for NRD alignment over 340 atoms). Backbone RMSD for alignment of the whole Edc1-Dcp1-Dcp2 complex is 2.5 Å over 856 atoms.

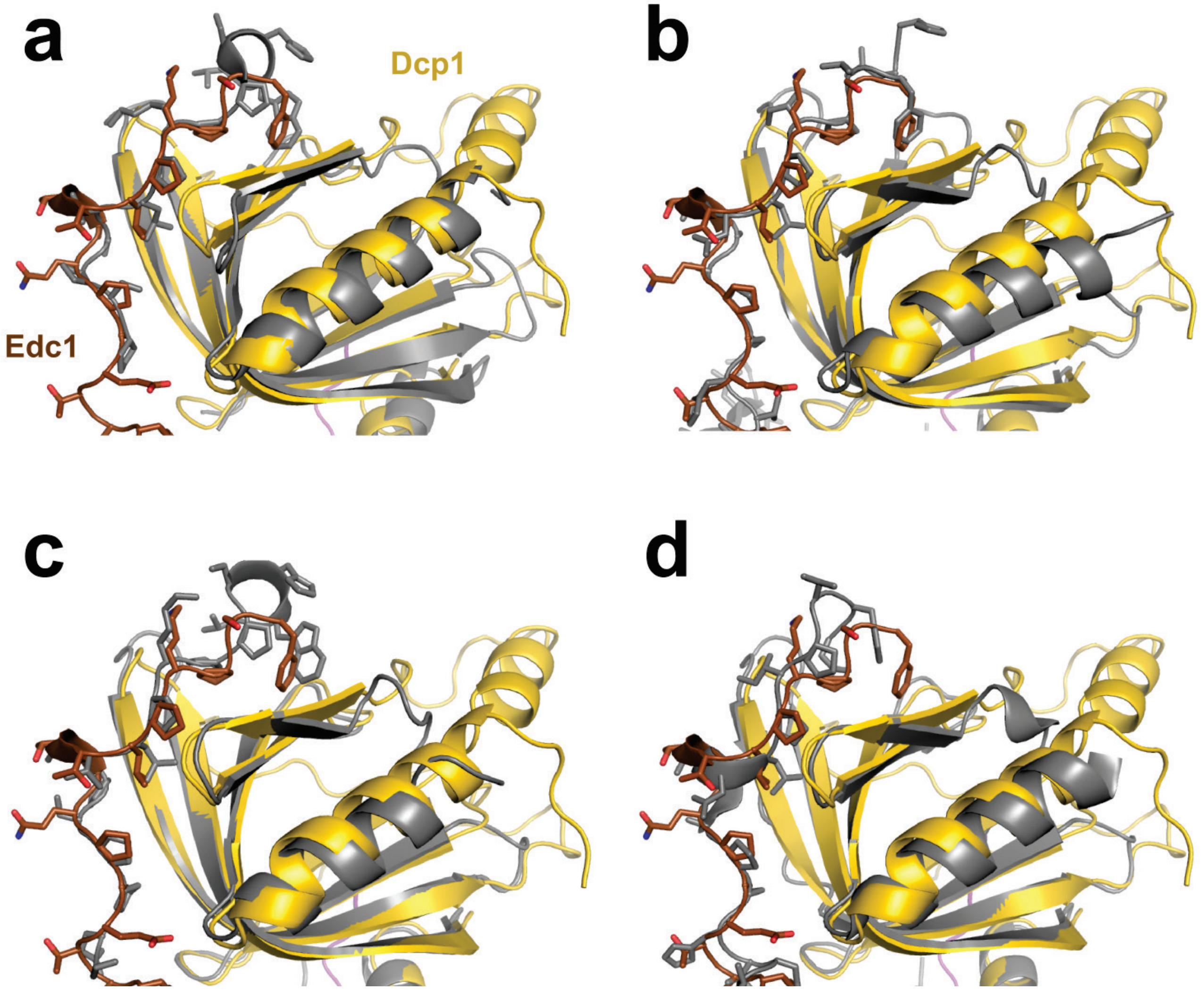

Supplementary Figure 2. Alignment of recent structures of Edc1-like peptides binding to Dcp1.

(a) Alignment of *Hs* PNRC2-Dcp1a structure (PDB 4B6H, gray) with *Kl* Edc1-Dcp1 from the structure presented in this paper (PDB 6AM0, colored); Dcp1 was used to align the structures with backbone RMSD of 2.38 Å over 488 atoms. **(b)** Alignment of *Sp* Edc1-Dcp1 structure (PDB 5J3T, gray) with *Kl* Edc1-Dcp1 from the structure presented in this paper (PDB 6AM0, colored); Dcp1 was used to align the structures with backbone RMSD of 2.59 Å over 448 atoms. **(c)** Alignment of *Hs* PNRC2- *Sp* Dcp1 structure (PDB 5KQ4, gray) with *Kl* Edc1-Dcp1 from the structure presented in this paper (PDB 6AM0, colored); Dcp1 was used to align the structures with backbone RMSD of 1.6 Å over 464 atoms. **(d)** Alignment of *Sp* Edc1-Dcp1 structure (PDB 5N2V, gray) with *Kl* Edc1-Dcp1 from the structure presented in this paper (PDB 6AM0, colored); Dcp1 was used to align the structures with backbone RMSD of 1.6 Å over 456 atoms.

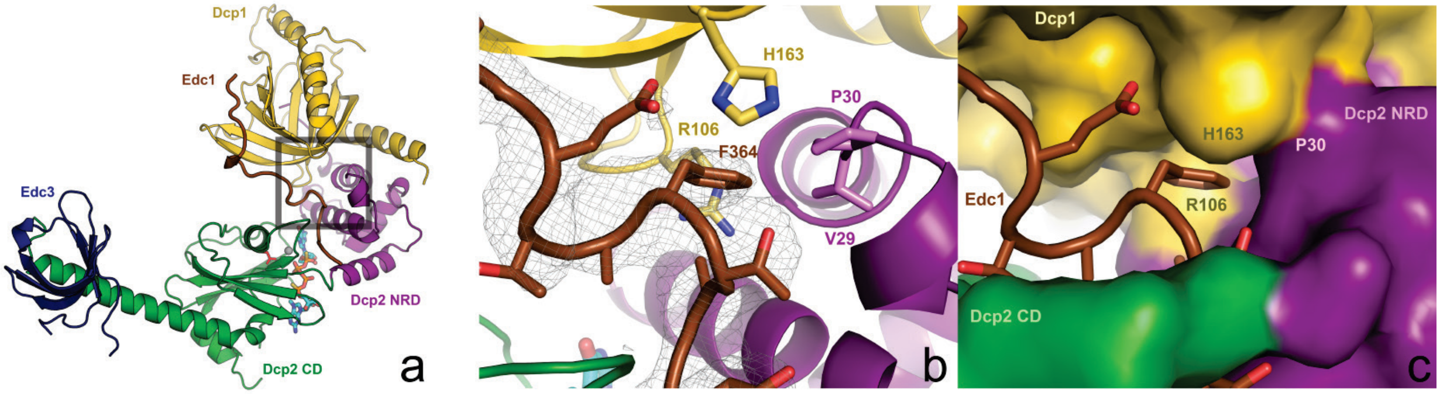

Supplementary Figure 3. *Kl* Edc1 Phe364 binding pocket at the Dcp1 – Dcp2 NRD interface.

(a) Overall *Kl* Edc1-Dcp1-Dcp2-Edc3 structure, with box showing Dcp1-Dcp2 NRD interface where Phe364 of the Edc1 activation motif binds. **(b)** Conserved residues on Dcp1 (yellow) and Dcp2 NRD (purple) that form the Edc1 Phe364 binding pocket are shown as sticks. **(c)** Surface view of the Phe364 binding pocket.

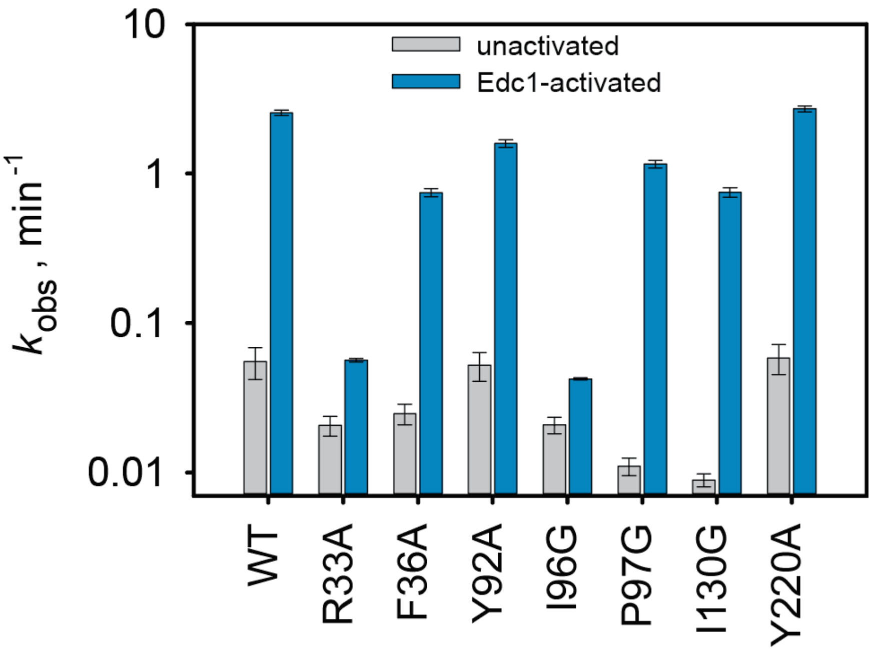

Supplementary Figure 4. Decapping activity of WT and Dcp2 mutants for *Sp* Dcp1-Dcp2 complexes and activated *Sp* Edc1-Dcp1-Dcp2 complexes.

Measured k_{obs} values for decapping a 355-mer capped RNA with *Sp* Dcp1-Dcp2(1-243) complexes (gray bars), or activated *Sp* Edc1(155-180)-Dcp1-Dcp2(1-245) complexes (blue bars). All mutations are on Dcp2. The Dcp1-Dcp2 concentration is 2 µM and Edc1 peptide is added in excess at 100 µM. Mutations of Dcp2 residues that contact the YAG activation motif of Edc1 in the active conformation have relatively small changes in decapping activity for the Dcp1-Dcp2 complex alone (gray bars); the largest change is the I130G mutation, which reduced decapping activity by ~6-fold in the unactivated complex. Upon addition of Edc1 peptide, most of the these Dcp1-Dcp2 mutant complexes are robustly activated by Edc1 by ~50-fold, and the smaller defects in decapping seen in the Dcp1-Dcp2 complex, such as with I130G, are rescued by addition of Edc1 and the complex is activated as in the WT case. The notable exceptions are the R33A and I96G mutations, which completely prevent activation of the Dcp1-Dcp2 complex by Edc1. Errors are standard error of fits to obtain k_{obs} values from two independent replicates.

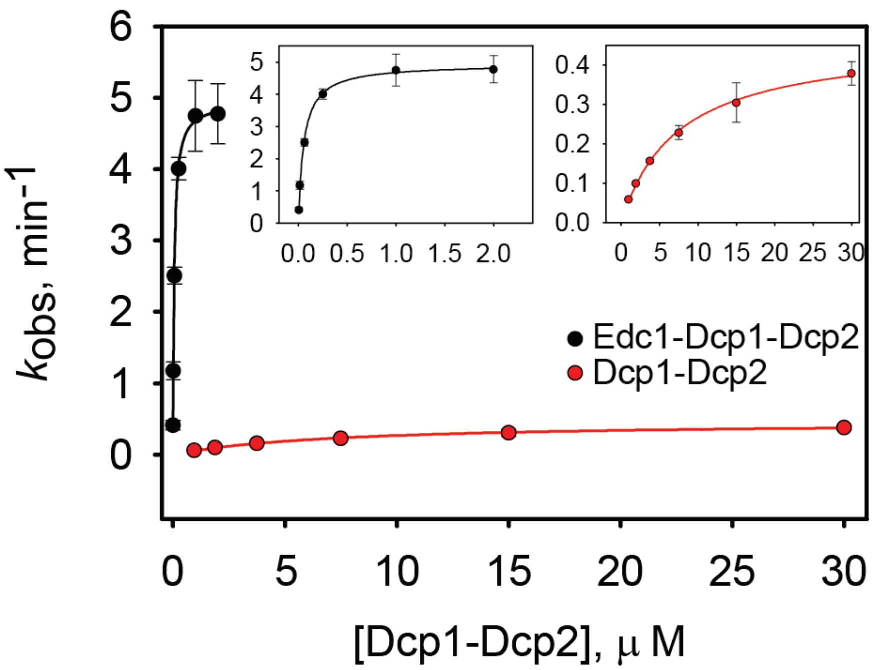

Supplementary Figure 5. *Sp* Edc1 peptide lowers K_M and increases k_{max}.

Plots showing k_{obs} versus Dcp1-Dcp2 concentration for Dcp1-Dcp2 complex with added *Sp* Edc1(155-180) peptide (50 μM peptide, black circles), or Dcp1-Dcp2 complex with no Edc1 activator (red circles). A radiolabeled, capped 29-mer RNA substrate is used in these experiments (G-RNA from Fig. 4; black circles are the same data as WT + G-RNA shown in Fig. 4). Insets show these same kinetic data rescaled for the individual data set. Upon addition of *Sp* Edc1 peptide, k_{max} is increased ~10-fold and K_M is decreased ~100-fold relative to the Dcp1-Dcp2 complex alone. See Supplementary Table 1 for K_M and k_{max} values. Errors on k_{obs} are s.d. of two independent replicates.

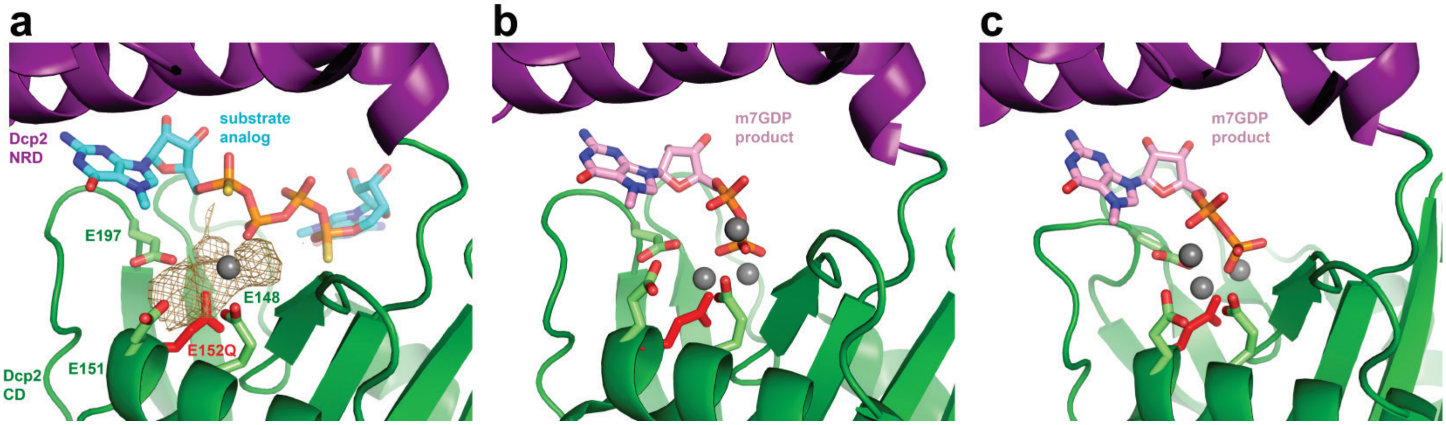

Supplementary Figure 6. Comparison of metal binding in the Dcp2 active site for substrate analog and product-bound structures.

(a) Dcp2 active site for *Kl* Edc1-Dcp1-Dcp2-Edc3 with substrate analog (PDB 6AM0). Dcp1 and Edc1 are omitted for clarity. Dcp2 NRD is purple, Dcp2 CD is green, F_o-F_c difference map shown at 2.0 σ in beige, Mg^{2+} are gray spheres, conserved Glu residues in the active site are shown as sticks, with the catalytic base Glu152 (mutated in this structure to Gln) shown in red. **(b)** Dcp2 active site for *Kl* Dcp1-Dcp2-Edc3 with m^7GDP product (PDB 5LOP). Colors and view as in (a). **(c)** Dcp2 active site for *Sp* Edc1-Dcp1-Dcp2 with m^7GDP product (PDB 5N2V). Colors and view as in (a).

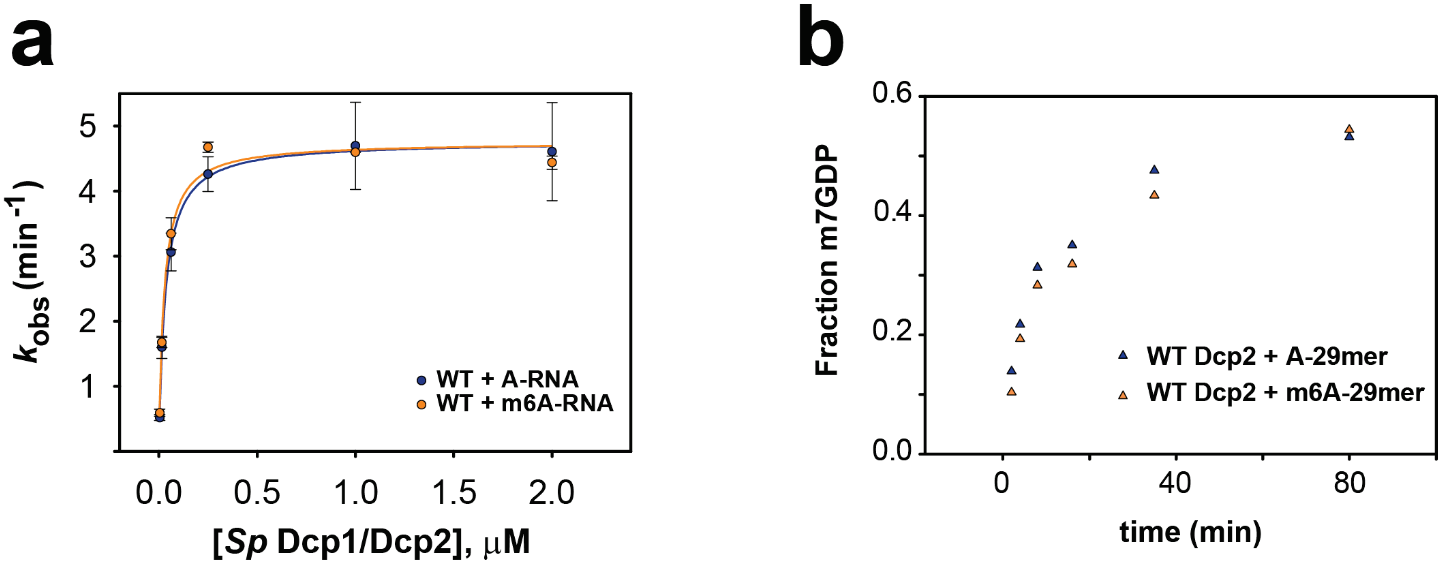

Supplementary Figure 7. Fission yeast decapping complex does not discriminate between A-RNA and m^6A-RNA.

(a) Plots showing k_{obs} versus Dcp1-Dcp2 concentration for Dcp1-Dcp2 complex with added saturating *Sp* Edc1(155-180) peptide (50 µM peptide) decapping a 29-mer radiolabeled capped RNA with A or m^6A as the first transcribed nucleotide (A-RNA or m^6A-RNA, respectively). The *S. pombe* Edc1-Dcp1-Dcp2 complex decaps A-RNA and m^6A-RNA with identical rates. Errors on k_{obs} are s.d. of two independent replicates. **(b)** Single time course showing *S. pombe* Dcp2 (20 µM) decapping A-RNA versus m6A-RNA. As with the activated Edc1-Dcp1-Dcp2 complex shown in (a), *Sp* Dcp2 alone does not discriminate between A-RNA and m^6A-RNA.

Supplementary Table 1. K_M and k_{max} values for *S. pombe* Edc1-Dcp1-Dcp2 and *Sp* Dcp1-Dcp2 complexes decapping a 29mer RNA substrate. Values and errors are obtained from the fits shown in Supplementary Fig. 5.

	k_{max} (min^{-1})	K_M (µM)
**Edc1-Dcp1-Dcp2**	4.94 ± 0.07	0.056 ± 0.004
**Dcp1-Dcp2**	0.47 ± 0.01	7.4 ± 0.5

Supplementary Table 2. K_M and k_{max} values for *S. pombe* Edc1-Dcp1-Dcp2 complexes (WT or Y220G) decapping 29mer RNA substrates with different nucleotides (G, A, or C) as the first transcribed nucleotide. Values and errors obtained from the fits of k_{obs} vs concentration data shown in Fig. 4a.

	k_{max} (min^{-1})	K_M (µM)
WT Edc1-Dcp1-Dcp2, G-RNA	4.94 ± 0.07	0.056 ± 0.004
WT Edc1-Dcp1-Dcp2, A-RNA	4.77 ± 0.05	0.032 ± 0.002
WT Edc1-Dcp1-Dcp2, C-RNA	3.10 ± 0.06	0.036 ± 0.003
Edc1-Dcp1-Dcp2(Y220G), G-RNA	6.5 ± 0.1	0.056 ± 0.005
Edc1-Dcp1-Dcp2(Y220G), A-RNA	7.03 ± 0.09	0.045 ± 0.003
Edc1-Dcp1-Dcp2(Y220G), C-RNA	6.4 ± 0.2	0.046 ± 0.006

Supplementary Table 3. Codon-optimized DNA sequences used in this study.

Construct	***E.coli codon-optimized DNA sequence***
K. lactis his-tev-Dcp1(1-188)-Dcp2(1-275, E152Q)-Edc3(1-66)	GAAAACCTGTATTTTCAGGGATCCATGTCAACGGAAACACTTGAAATCTATCGCAAAG<br>CCCTGAATTTCAATGTCATCGCGCGTTATGATCCAAAGATCAAGCAACTGTTATTTCA<br>CACTCCTCATGCGACCGTATATAAATGGGGAGATGATAACTGGAACAAGCTGGAGTA<br>CCAGGGCGTTTTAGCTATTTACTTGCGCGACGTTGGGGACAAAGAAGCAATCCTGCC<br>TGAGGTATCGTCATACGATGATACAATCACAGGACAGCAGTCGGAGGCTAATACGCC<br>CCATGTATTAACCGGGCACGACATCTATAACTACGGCTTAATTATCATGAATCGTATC<br>AACCCTGATAACTTTTCTCTTGCCATCGCACCCAACTCTGTTCTGAATAAACGCAAGT<br>TATTCGCGCCTAATCGTGAGGAGGAACTTGAGCCCATGAAGGTAGAAGTCCGTGAC<br>GATTTAGTGATGATTAAAACGCTGAAGAAGGAAGTCTATGGTATTTGGGTACATACAC<br>CTGAGGATCGCCAGAATATTTACGAACTGATCAAATATCTGTTGGAGAACGAACCCA<br>CAGATAGTTTCACATGACCATCTTAGTATATTAGTTAAGTATAAGAAGGAGATATACAT<br>ATGAGCTTGCCCCTGCTGCGCCCCTTCGAAACAGTTAGTTTGGAAAACGCAGTAGAA<br>GATCTTGTGGTTCGCTTCATTCTTAATGTTCCGCCTGAGGATTTGTCTACAGTTGAAC<br>GCGTTTTGTTTCACTTTGAGGAAGCGAGCTGGTTCTATACCGATTTTGTTAAGTTGAT<br>GAACCCGTATTTGCCAAATCTGAGCATTAAATCCTTTTCCAAAATCGTGATCGATATTT<br>GTCCATTAATTTGGAACTGGGACATTACGCCAGAAAATGCCTTAGTGAAATTCTCCAA<br>TTATAAAAAGACAATCCCGGTGCGTGGGGCCGCGATTTTCAATGACTCATTGAGCAA<br>GATTCTTCTTCTTCGTGGAATCAATAGCAAGCATTGGAGCTTCCCACGTGGAAAAATC<br>GGCAAGGATGAAGACGACGTGGCCTGCTGCATCCGTGAAGTAAAAGAGCAAACTGG<br>ATTCGATCTGACGGGCTTTATTGACGCGGACCAGTATGTGGAACGCAATATGAATGG<br>AAAGAATTTCAAAATTTTCTTAGTAAAGGGAGTCCCGGAGGATTTTGAATTTAAGCCG<br>GAACATAAGAACGAAATCCAGGCTATTGAATGGAAAGATTTCAAAAAGTTGTCAAAGG<br>CTATCACTAAAAACGAAGGATCAGCCAAAGTGTTTCTTGTAAATTCCATGATTCGTCC<br>ATTGTCCCTTTATGTCAAGAATGAAAAACGCGCCAAAGACGAAAACAAGTTAAAGCTG<br>TACGCAGAAGAACACCTGAAATCAATTTTAGGGCTTAACAAGAAGGAAAATAAGATCG<br>TCCTTGACGCGGGCCGTTGATCTAGAAAGAATTTTGTTTAACTTTAAGAAGGAGATAT<br>ACATATGTTAAATTTCAAGGGGTATCAGATCGAAATCGAATTAAAAGACGGTAAACGC<br>ATCACCGGGACACTGAAGCAGGTATCGCCAAAATCTTTAACACTGACTGATGCAGTA<br>TTTCAGGACGGTGGGGTCTCCCCCGTTTTCAAGATTAAAGCAGATAAACTGTACGAT<br>CTGAAGGTACTGAAGTTGCCCCCCAATGCTTAACGCACTCGAGCACCACCACCAC
S. pombe his-GB1-tev-Dcp1(1-127)	ATGTCGTACTACCATCACCATCACCATCACGATTACGATATCCCAACGACCGAAAACC<br>TGTATTTTCTGGGCGCCATGGAGTACAAGCTTATCCTGAACGGTAAAACCCTGAAAG<br>GTGAAACCACCACCGAAGCTGTTGACGCTGCTACCGCGGAAAAAGTTTTCAAACAGT<br>ACGCTAACGACAACGGTGTTGACGGTGAATGGACCTACGACGACGCTACCAAAACC<br>TTCACGGTAACCGAAATCCCAACGACCGAAAACCTGTATTTTCAGGGCGAATTCATG<br>GAAGACGAAAATATCCTGCGCAATGCTGTTAATCTGCAAGTACTGAAATTCCACTACC<br>CGGAAATCGAATCCATTATCGATATCGCGAGCCACGTTGCGGTATATCAGTTTGACG<br>TAGGTAGCCAGAAATGGCTGAAAACCTCTATTGAAGGTACCTTTTTCCTGGTAAAAGA<br>CCAACGTGCTCGCGTAGGTTACGTTATTCTGAACCGCAATTCTCCGGAAAACCTGTA<br>CCTGTTCATCAATCACCCGAGCAACGTACACCTGGTTGACCGTTATCTGATCCACCG<br>TACCGAAAACCAACATGTAGTTGGTCTGTGGATGTTCGATCCAAACGACATGTCTCG<br>CATCTTCAATATCGTTAAAGAAAGCCTGCTGCGTTAA

Supplementary Table 4. DNA primers used in this study. /5Phos/ denotes 5' phosphorylation.

Primer	***Description***	***DNA sequence***
Amp Kld1d2e3 fwd	Amplify K. lactis Edc1-Dcp1-Dcp2-Edc3 gblock shown in Supplementary Table 3	GAAAACCTGTATTTTCAGGG
Amp Kld1d2e3 rev		GTGGTGGTGGTGCTC
Lin pVFT for Kl fwd	Linearize pVFT KAN expression vector for insertion of above gblock	CGCACTCGAGCACCAC
Lin pVFT for Kl rev		GGATCCCTGAAAATACAGGTTTTC
R33A fwd	Sp Dcp2(R33A) mutation on Sp Dcp1-Dcp2	/5Phos/CGTTGAAGCACTTTGTTTTCAAATC
R33A rev		/5Phos/CTACTTTGCTCCTCTGCTGGTAAATTC
F36A fwd	Sp Dcp2(F36A) mutation on Sp Dcp1-Dcp2	/5Phos/CGTTGAACGACTTTGTGCTCAAATC
F36A rev	Use R33A rev primer	---
Y92A fwd	Sp Dcp2(Y92A) mutation on Sp Dcp1-Dcp2	/5Phos/GTATTCCTGTCCGCGG
Y92A rev		/5Phos/GAGTCTTAGCTCGTAAAAAATCATC
I96G fwd	Sp Dcp2(I96G) mutation on Sp Dcp1-Dcp2	/5Phos/GATATAAGACTCGTGGTCCTGTCCG
I96G rev		/5Phos/GTAAAAAATCATCAAACGCCTCTTCATG
P97G fwd	Sp Dcp2(P97G) mutation on Sp Dcp1-Dcp2	/5Phos/GATATAAGACTCGTATTGGTGTCCGCG
P97G rev	Use I96G rev primer	---
I130G fwd	Sp Dcp2(I130G) mutation on Sp Dcp1-Dcp2	/5Phos/CTAAGGGAAAAGGTGATAAAGATGAATC
I130G rev		/5Phos/GAAATCCCCAGCCGGATG
Y220A fwd	Sp Dcp2(I130G) mutation on Sp Dcp1-Dcp2	/5Phos/CCATTTTTGGCGCCATT
Y220A rev		/5Phos/GATTACCATGGCAAATTTGTTTTTC
Y220G fwd	Use Y220A fwd primer	---
Y220G rev	Sp Dcp2(Y220G) mutation on Sp Dcp1-Dcp2	/5Phos/GATTACCATGCCAAATTTGTTTTTC
